# 740. Healthcare-associated invasive *Staphylococcus aureus* among adults with prior COVID-19-associated hospitalization, 2020

**DOI:** 10.1093/ofid/ofad500.801

**Published:** 2023-11-27

**Authors:** Kelly A Jackson, Sydney Resler, Joelle Nadle, Susan Petit, Kimberly Yousey-Hindes, Susan M Ray, Ruth Lynfield, Kathryn Como-Sabetti, Carmen Bernu, Ghinwa Dumyati, Marissa Tracy, William Schaffner, Kadam Patel, Fiona P Havers, Holly M Biggs, Isaac See, Isaac See

**Affiliations:** U.S. Centers for Disease Control and Prevention, Atlanta, Georgia; Rollins School of Public Health at Emory University, Atlanta, Georgia; California Emerging Infections Program, Oakland, California; Connecticut Department of Public Health, Hartford, Connecticut; Yale School of Public Health, New Haven, Connecticut; Emory University School of Medicine, Atlanta, Georgia; Minnesota Department of Health, St. Paul, MN; Minnesota Department of Health, St. Paul, MN; Minnesota Department of Health, St. Paul, MN; New York Emerging Infections Program and University of Rochester Medical Center, Rochester, New York; University of Rochester Medical Center, Rochester, New York; Vanderbilt University Medical Center, Nashville, Tennessee; Centers for Disease Control and Prevention, Atlanta, Georgia; CDC, Atlanta, Georgia; U.S. Centers for Disease Control and Prevention, Atlanta, Georgia; U.S. Centers for Disease Control and Prevention, Atlanta, Georgia; U.S. Centers for Disease Control and Prevention, Atlanta, Georgia

## Abstract

**Background:**

COVID-19 caused a large proportion of hospitalizations in 2020. We describe how COVID-19-associated hospitalizations affected the epidemiology of healthcare-associated (HA) invasive *Staphylococcus aureus* (iSA) infections.

**Methods:**

The CDC Emerging Infections Program identified iSA cases and COVID-19-associated hospitalizations in adult surveillance area residents during March 1–December 31, 2020, from 9 counties in 6 states. iSA, HA (including hospital-onset [HO]) iSA, and iSA with prior COVID-19-associated hospitalization are defined in the Table. We used multivariable logistic regression to identify factors associated with COVID-19-associated hospitalization in persons with iSA infection and compared infection types and outcomes in iSA patients with and without prior COVID-19-associated hospitalization.

**Table.**
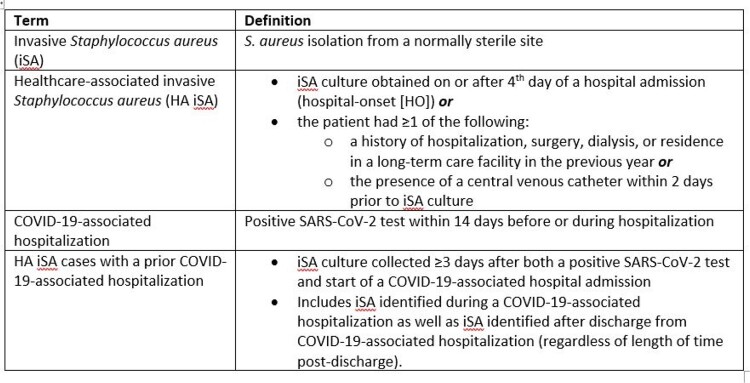
Definitions

**Results:**

Of 2,406 HA iSA cases reported, 146 (6.1%) had prior COVID-19-associated hospitalization. Most (58.9%) HA iSA cases with prior COVID-19-associated hospitalization were HO compared with 18.6% of other HA iSA cases (P< .01). The percentage of methicillin-resistant iSA cases was similar (HA iSA with prior COVID-19-associated hospitalization: 39.0% vs other HA iSA: 39.5%). In multivariable analysis, HA iSA cases with prior COVID-19-associated hospitalization more were often aged ≥65 years; of Hispanic ethnicity or Black race; had a central venous catheter, stay in a long-term care facility or long-term acute care hospital, and intensive care unit (ICU) admission before iSA culture; and less often had prior surgery and dialysis (Figure). The most common iSA-related diagnoses were bacteremia (HA iSA with prior COVID-19-associated hospitalization: 93.8% vs other HA iSA: 87.0%, P< .01) and pneumonia (32.9% vs 10.8%, P< .01). HA iSA cases with prior COVID-19-associated hospitalization more often were in the ICU after iSA culture (51.7% vs 26.6%, P< .01) and died (44.5% vs 13.8%, P< .01) vs other HA iSA cases.

**Figure.**
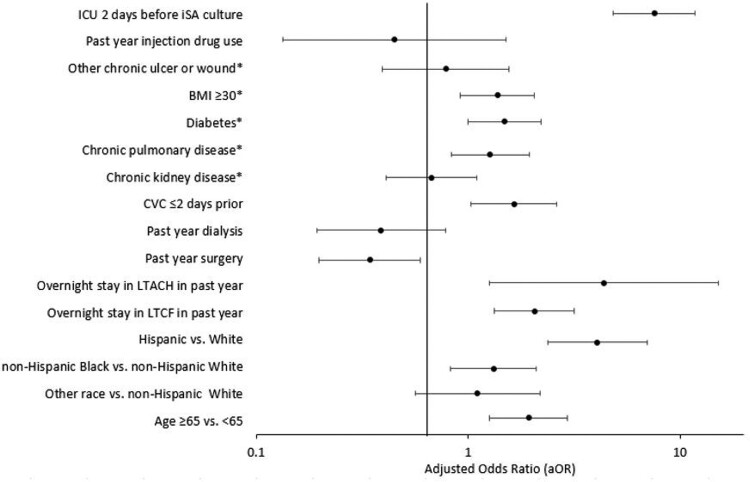
Multivariable logistic regression of demographic and epidemiologic characteristics associated with having a COVID-19-associated hospitalization, Emerging Infections Program data on healthcare-associated iSA cases from 9 US counties, March 1–December 31, 2020 *Underlying conditions noted in the medical record during the patients iSA-related hospitalization; ICU: Intensive care unit; BMI: Body mass index; CVC: Central venous catheter; LTACH: Long-term acute care hospital; LTCF: Long-term care facility

**Conclusion:**

HA iSA cases with prior COVID-19-associated hospitalization were commonly HO, followed intensive care interventions, and had higher death rates. Further exploration of iSA risk factors during COVID-19-associated hospitalizations might identify ways to prevent iSA infections and ensuing severe outcomes.

**Disclosures:**

**Ghinwa Dumyati, MD**, Pfizer: Grant/Research Support

